# Low signs of territorial behavior in the Eurasian otter during low-water conditions in a Mediterranean river

**DOI:** 10.1038/s41598-024-62432-1

**Published:** 2024-05-20

**Authors:** José Jiménez, Lucía Del Río, Pablo Ferreras, Raquel Godinho

**Affiliations:** 1 Instituto de Investigación en Recursos Cinegéticos (IREC, CSIC-UCLM-JCCM), Ronda de Toledo 12, 13071 Ciudad Real, Spain; 2 Centro de Investigação em Biodiversidade e Recursos Genéticos, InBIO Laboratório Associado, CIBIO, Universidade Do Porto, Campus de Vairão, 4485-661 Vairão, Portugal; 3 Departamento de Biologia, Faculdade de Ciências, Universidade Do Porto, 4169-007 Porto, Portugal; 4 BIOPOLIS Program in Genomics, Biodiversity and Land Planning, CIBIO, Campus de Vairão, 4485-661 Vairão, Portugal

**Keywords:** Aggregation, Density estimate, *Lutra lutra*, Mediterranean rivers, Pools, Spatial capture-recapture, Ecological modelling, Freshwater ecology

## Abstract

The Eurasian otter *Lutra lutra* is a territorial semi-aquatic carnivore usually found at low densities in rivers, coastal areas, and wetlands. Its diet is based on prey associated with aquatic environments. Mediterranean rivers are highly seasonal, and suffer reduced flow during the summer, resulting in isolated river sections (pools) that sometimes can be left with a minimal amount of water, leading to concentrations of food for otters. To our knowledge, this process, which was known to field naturalists, has not been accurately described, nor have otter densities been estimated under these conditions. In this study, we describe the population size and movements of an aggregation of otters in an isolated pool in the Guadiana River in the Tablas de Daimiel National Park (central Spain), which progressively dried out during the spring–summer of 2022, in a context of low connectivity due to the absence of circulating water in the Guadiana and Gigüela rivers. Using non-invasive genetic sampling of 120 spraints collected along 79.4 km of sampling transects and spatial capture-recapture methods, we estimated the otter density at 1.71 individuals/km of river channel length (4.21 individuals/km^2^) in a progressively drying river pool, up to five times higher than previously described in the Iberian Peninsula. The movement patterns obtained with the spatial capture-recapture model are not quite different from those described in low density, which seems to indicate a wide home range overlap, with low signs of territoriality.

## Introduction

Wildlife management and conservation require the understanding of ecological processes and demographic parameters^1^. A case in point is the description of the dynamics of carnivore territoriality^2^. Its intraspecific variation has been described in relation to food (quantity, predictability, distribution, quality, renewal rate, type, density, and accessibility) along other variables^3^. Maher and Lott hypothesized an inverted U-shaped relationship between the ecological variables and territoriality^3^. According to this hypothesis, we might find a decrease in intraspecific competition both under conditions of extreme food scarcity^4^ and higher availability of food, leading to increased tolerance towards conspecifics^5^. There are numerous descriptions of plasticity in social behavior (in both extremes of the inverted U-shape) as aggregations of usually solitary territorial predators that can be associated with situations of high prey availability^6^; e.g. wolverine (*Gulo gulo*)^7^, brown bear (*Ursus arctos*)^8^ and Iberian lynx (*Lynx pardinus*)^9^.

The Eurasian otter *Lutra lutra*, listed as a Near Threatened A2c species by the IUCN^10^, is a territorial top predator in aquatic ecosystems and is usually found at low densities^11,12^ compared to other terrestrial predators of similar size. The otter population in Spain is expanding and now occupies 59.9% of the Spanish peninsular territory, including dry areas with temporary water bodies as well as near large towns and cities^13^.

Eurasian otter typically occupies linear habitats, such as rivers or shorelines, and the number of evidential signs, such as feces (‘spraints’) or footprints, per linear kilometer, has been widely used as an index for comparing the relative abundance of otters at different spatio-temporal scales^14,15^. However, there is evidence of bias in the relationship between spraint abundance and otter numbers^16^. Other approximations have been used to estimate population density, such as: genetic non-spatial capture-mark-recapture methods^17^; direct count of individuals identified through molecular methods^18^; radio tracking data integrated with information from other data sources^19^; and direct observation^20^. In general, these studies agreed on the relatively low density of Eurasian otter populations compared to those reported for terrestrial mesocarnivores^21,22^ but this is also dependent on habitat specialization. Although the otter is generally considered to be a solitary territorial species^11,23^, a recent meta-analysis^24^ showed that in certain situations of high vegetation cover and prey availability, otter populations can exhibit other social patterns, including flexible territoriality and matrilineal groups. Quaglietta et al.^25^ found such patterns in southern Portugal, with some plasticity in social behavior. They described how opposite sex individuals could exhibit tolerance towards each other in important parts of their home ranges, including feeding, resting, and rearing sites. These authors also suggested that water shortage in summer could increase otters’ tolerance to conspecifics and force them toward mutual exploitation of aquatic areas. In the case of Mediterranean rivers, where otters behave as a more generalist predator compared to other otters in temperate rivers^26^, periodic situations of localized prey overabundance occur along low-water periods in spring–summer. This leads to a sequential exploitation of resources in isolated riverine pools (“pozas”), i.e., otters consume the available prey, before moving to another pool^27^. The importance of riverine pools for otters in Mediterranean environments during the dry season has previously been highlighted by Ruiz-Olmo et al.^28^.

To our knowledge, the otter population size and movements have never been quantified in this situation of low-water nor studied its spatial pattern. Spatially explicit capture-recapture (SCR) models provide a valuable tool for estimating a species’ density and population size^29^ to describe its biological underlying processes^30^. However, to our knowledge, the North American river otter *Lontra canadensis* is the only otter species for which SCR has been used^31^. The aim of our study was to describe and estimate the local otter population densities, in a situation of an isolated riverine pool drying by using genetic non-invasive sampling (gNIS) and SCR. Our hypothesis was that if prey concentrations increase as rivers dry to form isolated pools and territorial behavior relaxes, local otter densities would be expected to be higher than those described in continuously flowing river conditions. We compared the availability and use of prey to confirm that otters actually rely on residual pools for feeding^28^. We hypothesized that an increased tolerance between individuals under such conditions can be explained by kinship among part of the resident otters^23^.

## Material and methods

### Site description

**Figure 1:**
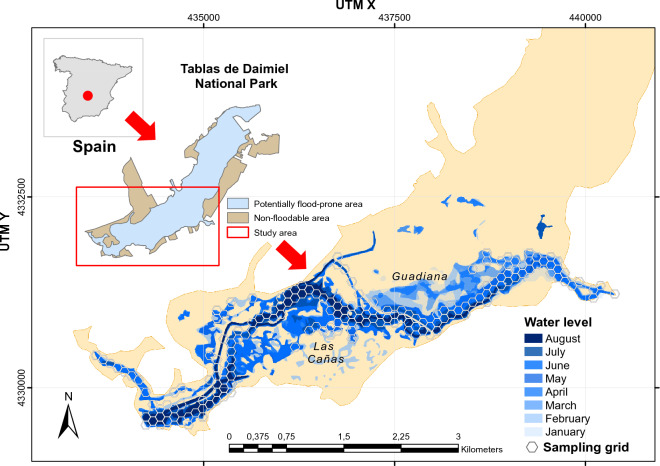
Map showing the location of the Tablas de Daimiel National Park (top left), the study area (red rectangle), and changes in the flooded area between January and August 2022. The hexagonal grid was used to divide the sampling area into sampling cells, the samples in each cell being assigned to its centroid, to generate 'detectors' for use in the spatial capture-recapture model. Created using ArcGIS^63^.

The study was carried out in the Tablas de Daimiel National Park (TDNP; Ciudad Real, central Spain) (39º7′59.59″N 3º42′56.18″W), located on the central Spanish southern plateau (Fig. 1). The park was originally a permanent wetland formed by the confluence of the permanent fresh waters of the Guadiana River, arising from the upwelling of the Western Mancha aquifer system, and the more seasonal saline waters of the Gigüela River. Since the 1980s, overexploitation of the aquifer for irrigation has radically reduced the flow of the Guadiana River, and the park has become mainly dependent on water from the Gigüela River, which rarely floods the TDNP. In addition, the park is affected by inputs of poorly treated effluent from wastewater treatment plants along the Gigüela and Guadiana Rivers^32^. Following a brief period (2010–2014) of complete inundation in TDNP, which was a consequence of the partial recovery of the aquifer^33^, since 2018 the TDNP has again suffered a loss of water input from the Guadiana due to overexploitation, a situation that persists today. Consequently, the hydrological conditions have become markedly seasonal and dependent upon both irregular inflows from rivers and artificial inflows of well water controlled by TDNP managers. This results in a flood peak between late winter and mid-spring and minimum water levels during late summer and early autumn. At the landscape level, over-exploitation of water affects river connectivity for long periods (even several years) and exacerbates the fragmentation of animal populations. The nearest downstream wetland with otters is El Vicario reservoir on the Guadiana River, 19 km away. Upstream, otters occur on the Azuer River and the Vallehermoso reservoir, 65 km away. In the year of this study (2022), all rivers within a 50 km radius of TDNP were dry.

The National Park hosts a population of Eurasian otters, previously estimated by Jiménez et al.^34^ using unmarked-SCR^35^ in spring 2013 at 0.557 (SD: 0.317) individuals/km^2^, or 11.14 (SD: 6.35) individuals in 1800 ha of flooded area that remained constant throughout 2013. The ichthyofaunal diversity of the TDNP has changed radically over the last 100 years. The community of small native cyprinids (of the genera *Barbus*, *Squalius*, *Chondrostroma*, and *Cobitis*), has been replaced by non-native species during the twentieth century such as common carp *Cyprinus carpio*, goldfish *Carassius auratus*, pumpkinseed sunfish *Lepomis gibbosus* and eastern mosquitofish *Gambusia holbrooki*, and more recently (2010) black catfish *Ameiurus melas*. In terms of biomass, the fish community is currently dominated by non-native species, mainly common carp and goldfish^36^. During a desiccation process in 1999, the biomass of fish in the TDNP was estimated to be 5000–6000 kg ha^−1^ of carp, 1300–1700 kg ha^−1^ of pumpkinseed sunfish, and approximately 115 kg ha^−1^ of mosquitofish^37^. These figures are 100 times higher than those reported in a Mediterranean environment in Australia^38^. Non-native American crayfish *Procambarus clarkii* was introduced into TDNP in the 1980s, and is currently abundant^39^. Biomass estimates for American crayfish in TDNP (not during a drying process) ranged from 819–929 kg ha^−1^ in 2000^40^. To understand its importance in this context, it is worth noting that the American crayfish has been identified as one of the main prey items of the otter in its Iberian range distribution^41–43^.

### Data collection

We searched the study area looking for otter spraints. Sampling was carried out by a single researcher over 12 days in 2022, between April 18–29, and May 9–12. Transect sampling was conducted on foot and by boat over a distance of 79.4 km. Sampling covered the flooded area up to the February flood-line (317 ha). The sampled area farthest from the shore had dried out in the previous two months. The oldest samples could therefore be from February, that was used as a conservative reference for density calculation. By the end of May, the flooded area had fallen to 185.4 ha. Sampling was performed primarily by walking along the banks and small islands that otters commonly used for marking and resting, and by using boats to access small islands and by searching for emerging logs and stones in the flooded areas. All transects were recorded using a GPS device (Garmin^©^ ETREX 32X). The locations of individual spraint samples were geo-referenced using a GPS handheld device and samples were individually preserved in vials containing 96% ethanol^44^.

### Genetic analysis

Otter individuals were identified through the analysis of the DNA extracted from the fresh collected samples. All pre-PCR operations were conducted under sterile conditions and positive air pressure in dedicated laboratory rooms. DNA extraction from spraint samples followed the GuSCN/silica protocol^45^. Extracts were further filtered for potential PCR inhibitors using pre-rinsed Microcon® YM-30 Centrifugal Filter Units (Millipore, Burlington, VT, USA). Possible DNA cross-contamination was monitored using negative controls. Individual DNA identification was achieved using a set of 20 microsatellites specifically developed for the Eurasian otter^46,47^. Details of the loci are shown in the Supplementary Information, Table S1. Amplification of markers was performed using a pre-amplification protocol^48^. Markers were pooled into four multiplex sets, with four to six markers each, and amplified using the QIAGEN Multiplex PCR Kit (Qiagen. Hilden, Germany) following the manufacturer’s instructions. (Qiagen. Hilden, Germany). Details of the thermocycling conditions are given in the Supplementary Information, Table S2. Four amplification replicates per sample were performed, always including negative controls to monitor possible DNA cross-contamination. The PCR products were separated by size using an ABI3130xl genetic analyzer (Thermo Fisher, Waltham, MA, USA). Alleles were scored against the GeneScan500 LIZ size standard, using GENEMAPPER 5.0 (Applied Biosystems, https://www.thermofisher.com/order/catalog/product/4370784) and checked manually. The sex of individuals was identified using the LutSRY marker^49^ genotyped within one of the multiplex systems (Supplementary Information, Table S1).

Consensus genotypes over the four replicas were assembled manually, following Godinho et al.^50^. Heterozygous genotypes were accepted if the same genotype was observed in two independent PCRs. Homozygous genotypes were accepted if the genotype was observed in three independent PCRs. Consensus genotypes with > 14 loci were used in the further analysis. Mean allelic dropout and false allele rates across loci were estimated using GIMLET 1.3.3^51^. Identical genotypes were filtered using GenAlEx 6.5^52^. The same software was used to estimate the cumulative probability of identical genotypes being shared by chance (probability of identity, PID and PIDsibs) for the 20 loci in the dataset and for 10 datasets of 14 randomly selected loci (minimum number of loci genotyped in our dataset).

To infer potential parentage and sibling relationships among identified individuals we used the full-likelihood method implemented in Colony v2.0.6.6^53^. We allowed for male and female polygamy and assumed locus-specific allelic dropout rates. All individuals were considered as potential offspring and all males and females as potential fathers and mothers, respectively, as no a priori information was available. Allele frequencies were calculated from the data.

### Otter population size estimates

As an analytical method, SCR provides information about the study population size and its space use. The standard SCR model^3^ assumes that individual activity centers (ACs) $i = 1, 2,\dots ,N$ are distributed over a region or state space $S$ and that individuals were sampled by our detector array within $S$. The distribution of individual ACs $s=({s}_{x},{s}_{y})$, was described in our study by a homogeneous point process, such that ${s}_{i}\sim Uniform\left(S\right)$. The ACs are latent variables to be estimated by the model given the detector-specific events for the *n* detected individuals at detectors $j=\text{1,2},\dots J$ with locations ${x}_{j}=\left({x}_{{j}_{1}},{x}_{{j}_{2}}\right)$. Assuming that detection frequencies are a decreasing function of the distance ${d}_{ij}$ between individual ACs ${s}_{i}$ and a detector location ${x}_{j}$, the expected detection rate (1) can be defined as:

1 $${\lambda }_{ij}=\lambda \left({s}_{i},{x}_{j}\right)={\lambda }_{0}\times exp\left(-\frac{{d}_{ij}^{2}}{{2\sigma }^{2}}\right)$$

where ${\lambda }_{0}$, the basal detection rate, is the expected detection rate when ${d}_{ij}=0$, indicating direct overlap of an AC with a detector; and $\sigma$ is the scale parameter of the half-normal detection function, which could be considered as a descriptive parameter for the movement of the target species. As our sampling was not characterized by a set of discrete “trap” locations, but by linear sampling, the sampled space was segmented into cells, assigning to the centroid of each cell all the samples collected in the same cell. We used a hexagonal cell grid over the sampled area. Hexagons have a low perimeter-to-area ratio, which reduces the sampling bias associated with edge effects due to grid shape. These hexagons’ centroids are hereafter referred to as "traps". The corresponding sampling effort per cell was used as a covariate of the baseline probability of detection of each trap. Many studies using gNIS and SCR have used this approach of discretizing the sampled area^44,54,55^. Although assigning the aggregated samples to centroids as 'detectors' may reduce the precision of the parameter estimates, Milleret et al.^56^ showed that SCR models using Poisson or partially aggregated binary observation models estimated abundance with low bias when the distance between centroids was small in relation to the movement of the target species. They recommended using a distance of less than $1.5\sigma$ between traps (centroids). We used a cell size of 1 ha (distance between centroids < 110 m), a very conservative value compared with the $\sigma$ values previously calculated for otters (unpublished data [1874 SD: 84 m]). One hundred and eighty-one hexagonal cells were used, with an average sampling effort of 439 (SD: 443) m/ha (Figure S1). In our study, the SCR model was applied to data with a single sampling occasion ($K = 1$) by collapsing the data from different days, since the complete sampling of the study area required several days of fieldwork and only a small part of the study area could be sampled each day. The model parameters were identifiable if several animals could be detected several times (and in several traps) to allow the estimate of $\sigma$ and ${\lambda }_{0}$ values^30^. Single sampling has been often used in SCR with genetic non-invasive sampling^44,54,55^.

We used the random thinning spatial capture-recapture (rt-SCR) model^54^, which is an SCR model that utilizes encounters of samples of the target species of both known and unknown identity with a natural mechanistic dependence between samples arising from a single observation model (sample collection and genotyping). Individual identification information can be lost in capture-recapture processes (e.g., genotyped samples without individual identification in gNIS). The process of assigning individual identities to samples in capture-recapture methods can be conceptualized as a random thinning process, where samples lose their individual identities at random, with a probability $1-\theta$. This process produces two types of data sets, one with individual identities, and another without individual identities. The rt-SCR model uses a sub-model for individual identification ${y}_{ij}^{ID}$ (2), conditional on the true encounter frequencies ${y}_{ij}^{true}$, assuming:

2 $${y}_{ij}^{ID} \sim Binomial\left({y}_{ij}^{true},\theta \right)$$

The individual identities of unrecognizable encounter frequencies ${y}_{ijk}^{noID}$ are then latent and ${y}_{ij}^{noID}={y}_{ij}^{true}-{y}_{ij}^{ID}$. For the unidentified samples, only the trap counts (counts in cells here) summed across captured individuals, ${nnid}_{jk}={\sum }_{i=1}^{N}{y}_{ijk}^{noID}$ can be observed. Thus, the same individual could be in both encounter histories—identified and not—in the same cell. Also, individuals with unidentified samples are not required to also be in the set of identified samples. This model was fitted using R^57^ and NIMBLE^58^ with a custom Metropolis-Hasting update for ${y}_{ij}^{true}$ that obeys the constraint ${y}_{ij}^{noID}={y}_{ij}^{true}-{y}_{ij}^{ID}$. By including non-ID samples that otherwise would be discarded, this model can improve density estimation for non-invasive sampling studies^48^.

In our sampling, many traps (cells) registered no detections (e.g., those in the water), and those that did (e.g., on the shoreline) had more detections than predicted by the model. Further heterogeneity arose from the difference in detectability between the samples collected on foot and by boat. We addressed this heterogeneity by adding a random effect (3) to the basal detection rate of each trap, following a common normal distribution:

3 $${\varepsilon }_{j}\sim Normal\left({\mu }_{0},{ \sigma }_{p}^{2}\right)$$

where ${\mu }_{0}$ is the log scale mean with a variance ${\sigma }_{p}^{2}$. Adding this term to the model takes account of trap-specific variability that could not be assigned to known sources of detection heterogeneity. Thus, the basal detection rate in each cell is a function of effort (${L}_{j}$: total length of sampling in any one cell) and random effect ${\varepsilon }_{j}$:

4 $$log\left({{\lambda }_{0}}_{j}\right)=\beta \cdot {L}_{j} + {\varepsilon }_{j}$$

We compared (1) the null model, (2) the model with ${L}_{j}$ as covariate in baseline detection rate (${\lambda }_{0}$) and (3) the model with ${L}_{j}$ as covariate and a random effect (${\varepsilon }_{j}$) in baseline detection rate, using the Widely Applicable Bayesian Information Criterion (WAIC)^59^. We hypothesized that otter ACs would be associated with flooded areas^21^ and constructed a habitat availability matrix for the SCR model using the R package, makeJAGSmask^60,61^ to restrict the analysis to the flooded area in February (see R + Nimble code in https://zenodo.org/records/10397199). This allowed us to measure otter densities relative to the flooded area (individuals/km^2^) and, as a derived parameter, population size and densities per linear kilometer of river. The linear reference for calculating the density per kilometer is the 7.8 km length of the river channel. The only deep zone (> 25 cm) was the Guadiana channel ("La Madre"). The rest was a muddy area that dried up during the study period.

The overlapping ACs probabilities made it difficult to visualize the density of the ACs from the model^3^ in a common raster plot. We chose to depict the probability of the ACs of each individual in a spatial plot of our model outcomes by constructing a contour map of the Empirical Bayes posterior distribution of the AC^30^ for each individual, using the 2D kernel density estimator with the *kde2d* function from the MASS^62^ package in R. We calculated the ACs as the points with the highest posterior probability. From these locations, we also calculated the average distance from each AC to the next nearest AC, considering either all individuals or only males (with more pronounced territoriality^11^). Finally, the dispersion-clustering pattern of the ACs was examined using ArcGIS^63^ software. We used the Average Nearest Neighbor (ANN) tool to measure the distance between each AC and its nearest neighbor's AC. If the average distance is less than the average for a hypothetical random distribution, the distribution of the ACs being analyzed is considered clustered. If the average distance is greater than a hypothetical random distribution the ACs are considered dispersed.

Posterior probabilities were calculated using three independent Markov chain Monte Carlo (MCMC) calculations, with 250,000 iterations each, adding a burn-in of 10,000 iterations, and thinning by five. We assessed the MCMC convergence and mixing by visual inspection of the trace plots and then calculated the Gelman-Rubin statistic (R-hat < 1.1)^64^ using the coda package in R^65^. For all parameters we calculated the posterior means for point estimates and 95% percentiles for the Bayesian credible intervals. We tested the goodness-of-fit (GoF) of the model using the approach suggested by Meredith^66^ as previously used by Jiménez et al.^67^ with three statistics to evaluate the observation model: (i) total number of detections; (ii) number of individuals detected; and (iii) total number of detectors visited, which is related to detector performance. For the GoF test we generated simulated detection data for all *M* individuals (including data augmentation) in rows and *J* columns for the detectors using the Markov chain Monte Carlo (MCMC) process. We plotted the observed and posterior predictions for each statistic, and we calculated the Bayesian *p-*value (used to measure the dissimilarity between observed data and model-predicted data). 

### Prey consumption

One hundred and one spraints collected during the sampling process was analyzed to determine the otter diet. Spraints were examined in the laboratory using identification guides to determine the prey species present in each sample. Identification was made macroscopically using a binocular microscope (Digital microscope MUSTOOL G600, ID: 1,152,799, 1-600x)^41,68^. Identifications were made at the lowest possible taxonomic level, generally species. The minimum number of individuals of each prey type in each sample was estimated from the number of diagnostic hard parts (pleopods for American crayfish and mainly operculum for fish). The importance of each prey category in the diet was estimated from its minimum number of prey items (n) in each sample, its frequency of occurrence (FO) (number of occurrences of a given item as a percentage of the total number of spraint samples) and its relative frequency of occurrence (RFO) (number of occurrences of a given item as a percentage of the total number of occurrences of all prey items)^69^. We were interested in the consumption of different prey species. If consumption of overabundant species was high, this could be indicative of exploitation of these prey concentrations^28^.

## Results

### Sampling, individual identification and parentage inference

A total of 251 whole or partial spraints were collected. Because the age of a spraint has been shown to be critical for genotyping success^70,71^, only the 120 freshest spraints were selected for genotyping.

We obtained 71 otter genotypes from the 120 freshest samples (59.2% success), corresponding to 13 different individuals observed in 29 cells. The observed sex ratio was 1:1.6 (5 females: 8 males). The average number of recaptures per individual was 5.46, with a range of 1–23. Of the ID-spatial recaptures (the same otter detected in different cells), one otter was detected in 14 cells, one in 10 cells, one in 6 cells, one in 4 cells, three in 2 cells, and six in one cell. We also used as data in rt-SCR model 49 non-ID otter genotypes (see R + Nimble code in https://zenodo.org/records/10397199). The average genotyping error rates across loci were 36.6% for allele dropout and 0.3% for false alleles (rates per locus are given in the Supplementary Information, Table S3). The estimated Probability of Identity for the dataset provided high confidence in the identification of individuals, with PID = 1.82 × 10^–10^ and PIDsibs = 1.23 × 10^–4^^72^. When 10 datasets of 14 randomly selected loci are used to calculate these statistics, the range of values observed varied between 7.18 × 10^–6^ and 1.11 × 10^–7^ for PID and 3.44 × 10^–3^ and 6.55 × 10^–4^ for PIDsib, confirming the accuracy of the dataset to differentiate individuals using a minimum of 14 loci, based on the reasonably accepted thresholds of 0.0001 and 0.01 for PID and PIDsib, respectively^72^ (Table S4).

**Figure 2:**
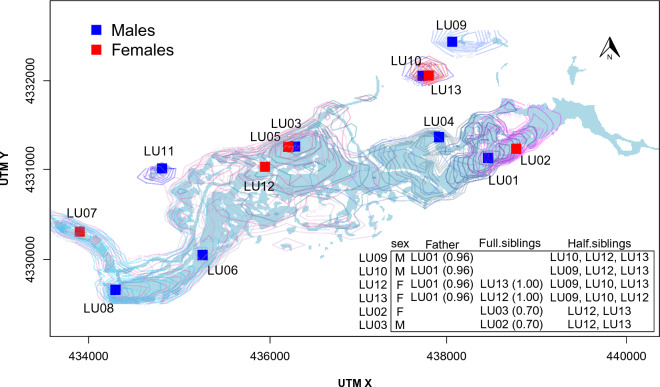
Local maxima for each individual otter *Lutra lutra* activity center, calculated from contour maps of the posterior distribution of activity centers in the Tablas de Daimiel National Park (Spain) in 2022. Table show direct familial relationships inferred among the 13 individuals. The six offspring are listed in the first column, along with their sex, father (when observed), full-siblings, and half-siblings. The probability of the relationship is given in parentheses for parent–offspring and full-siblings (see also Supplementary Information S2–S6, and Figure S7). Created using R^56^.

Seven of the 13 otters identified have a high probability of having a direct familial relationship among them. We observed two pairs of full siblings that share the same unsampled mother. Furthermore, the male LU01 was inferred as the only parent in the dataset, with four offspring, including one of the full-sibling pairs observed (Fig. 2 and Figure S7).

### Population size estimate

**Figure 3:**
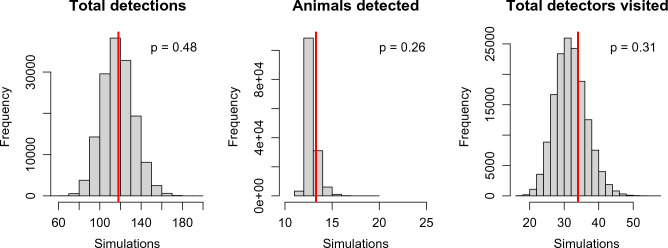
Histogram plots of observed values (red vertical lines) and posterior predictive values for the random thinning-spatial capture-recapture model. In each plot the Bayesian *p* value is shown (top right). Created using R^56^.

The model including a random effect by trap ${\varepsilon }_{j}$ was selected (WAIC = 159.4) over the null model (WAIC = 165.2) and the model with ${L}_{j}$ as covariate for the baseline detection rate (WAIC = 161.9). The top rt-SCR model estimated the population size at 13.33 (SD: 0.61) individuals (Table 1), which was very close to the 13 different individual genotypes obtained by the molecular analysis. The density relative to the flooded area in February was 4.21 individuals/km^2^ on an area basis, or 1.71 individuals/km of river channel length (Fig. 1). The density of the ACs was fairly uniform over the study area, with a higher concentration in the central area, which corresponded to the deepest parts of the river (Fig. 2, Figs. S2–S6). The half-normal scale parameter ($\sigma$) descriptive of movement was estimated at 1087 m (SD: 127.7) (Table 1). Hence, using the relationship from Royle et al.^73^, 95% of the movement outcomes were within 2662 m (SD: 312) from the center of an individual otter’s home range. The GoF was adequate for the three statistics studied (Fig. 3).

**Table 1:** Posterior summaries of the parameters from the random thinning spatial capture-recapture model used to estimate the otter *Lutra lutra* population in the Tablas de Daimiel National Park (Spain).

	Mean	SD	q2.50%	q50%	q97.5%
$\widehat{N}$	13.331	0.613	13.000	13.000	15.000
${\widehat{D}}_{[s]}$	4.205	0.193	4.101	4.101	4.732
${\widehat{D}}_{[L]}$	1.709	0.079	1.667	1.667	1.923
$\widehat{\psi }$	0.276	0.062	0.162	0.273	0.406
$\widehat{\beta }$	1.649	0.247	1.223	1.629	2.182
$\widehat{\sigma }$	1.088	0.128	0.869	1.077	1.370
${\widehat{\mu }}_{0}$	− 4.632	0.515	− 5.761	− 4.588	− 3.747
${\widehat{\sigma }}_{p}$	1.939	0.344	1.369	1.905	2.704
$\theta$	0.600	0.045	0.511	0.601	0.686

$\widehat{N}$ is the otter population size estimate in the flooded area in February 2022;${\widehat{D}}_{[s]}$ and ${\widehat{D}}_{[L]}$ are the density estimates (individuals/km^2^, and individuals/km of river length, respectively); $\widehat{\psi }$ is the parameter for data augmentation; $\widehat{\sigma }$ (in km) is the half-normal scale parameter describing the rate at which the detection probability declines as a function of distance from the detector; $\widehat{\beta }$ is the parameter for sampling effort in the baseline detection rate; ${\widehat{\mu }}_{0}$ and ${\widehat{\sigma }}_{p}$ are the random effects hyperparameters of the baseline detection rate. The mean and SD are shown for all parameters.

Using the ANN ArcGIS tool, the average distance between nearest individual's ACs (regardless of sex) was 465 m and the average distance between male ACs was 899 (Fig. 2). Significant evidence for dispersed ACs was recorded (ANN = 1.92, *p* < 0.05, and ANN = 2.03, *p* < 0.01 for all individuals and males, respectively).

### Prey consumption

The macroscopic diet study was based on 101 spraint samples. The most common prey item in the diet was the American crayfish (RFO 59.4%), followed by carp (12.9%), and goldfish (10.6%) (Table 2).

**Table 2:** Composition of the otter *Lutra lutra* diet as revealed by examination of spraints (n = 101 spraints) in the Tablas de Daimiel National Park.

Group	N	FO	RFO
Arthropods			
*Procambarus clarki*	60	59.41	57.69
Other arthropods	2	1.98	1.92
Fishes			
*Ameiurus melas*	5	4.95	4.81
*Carassius auratus*	11	10.89	10.58
*Cyprinus carpio*	13	12.87	12.50
*Lepomis gibbosus*	2	1.98	1.92
Undetermined fish	10	9.90	9.61
Reptiles	1	0.99	0.96

Frequencies of occurrence (FO), number (n), and relative frequency of occurrence (RFO) of the various diet categories.

## Discussion

Our results show a static scenario from February to April–May, this being the period over which we were able to attribute spraints collected 100–120 m from the river shoreline. The otter population density in 2022, in a progressively desiccating riverine pool in the TDNP, was 1.71 otters/km, much higher than that described for this species along freely flowing rivers, albeit using different methodologies. For instance, Quaglietta et al.^19^ reported an otter population (including males, females and juveniles) of 0.13–0.27 ind/km and an adult density of 0.07–0.14 ind/km in southern Portugal using radio-tracking, while Sittenthaler et al.^16^ estimated 0.16–0.28 otters/km along the Danube River (Austria) and Lerone et al.^18^ estimated 0.152 otters/km along the Sangro River (eastern central Italy), both using non-invasive genetic identification, the former also applying non-spatial capture-recapture models. Previously, we found a density of 0.308 (SD: 0.038) individuals/km using SCR along a river with circulating flow in the province of Seville (southern Spain), with a *σ* of 1874 km (unpublished data). Hájková et al.^74^ reported 0.49–0.96 otters/km^2^ in the Czech Republic, in a complex habitat composed of fishponds, channels, pools, marshes and river, with patchily and abundant food resources, similar to the TDNP in a flooded situation. All reference values, including those that recorded the entire otter population (adults and juveniles) are lower than those estimated in our study. When comparing the density per unit area (Table 1) with previous studies in the TDNP^34^, we found a much higher density in 2022. The level of kinship observed in the population studied (Fig. 2 and Figure S7) could partially explain the apparent relaxation on territoriality, as described in other carnivores^75^. This result would fit the kinship hypothesis^76^, which suggests that there are fitness benefits for individual animals that tolerate or even cooperate with related conspecifics. The abundance and natural concentration of prey in the study area may favor a longer staying of cubs with the mother and/or a longer staying together of cubs. However, it’s also worthy to notice that four non-related otters (among which 3 males) occupy the peripheral western portion of the study area, away from related otters (mostly concentrated at the eastern periphery). The higher tolerance among non-related otters may favor a staying reproductive tactic of males, with secure access to females concentrated in the habitat where preys are abundant, even if this requires them to be tolerant with other neighboring males. Oddly, while the density estimates in this study were much higher compared with other studies, the movement parameter $\sigma$ estimated from the SCR model was in close agreement with home range information under circulating flow conditions. For instance, Quaglietta et al.^19^ found that individual otters can occupy a range of 3.71–7.80 km of river length. This indicates a range of movement around their centers of activity of 1.86–3.90 km, nicely within the calculated value of 95% of movement outcomes (2.66 km) given by the rt-SCR model in our study. Even at the high density observed in this study, otter movement distances are large. The distance between the closest otter ACs, their spatial arrangement and multimodality (Figs. S2–S6), and the otter movements (as indicated by the *σ* values) imply that the areas of otter activity overlap. Our findings could indicate a poorly developed territoriality (as shown in Fig. 2 and Figs. S2–S6) and an increase in otter tolerance of conspecifics, even though some degree of territoriality still occurs. ACs appear to be distributed fairly evenly in males across the area. This indicates that territoriality is not completely abandoned, but is modified in the sense of non-exclusive utilization of territories. As the rivers in the TDNP dry out and otters are forced to aggregate, they mainly consume the most abundant prey in the park, namely American crayfish, common carp, and goldfish^32,37,40^. Although we lack estimates of American crayfish biomass in a situation of progressive desiccation, prey use by otters, as assessed by spraint analysis, is related to its availability, suggesting that otters tend to exploit fish and crayfish crammed in the residual waterbody. We would expect the aggregation to continue as long as the cost–benefit balance in the consumption of these prey species remains favorable^77^. It is possible that the aggregation density we have described would be even higher by the end of the summer, when the flooded area had shrunk to its minimum.

Although the river desiccation process described here is partly caused by the overexploitation of the aquifer that feeds the TDNP, the drying-up of rivers—even to the point of complete desiccation—is a common dynamic in Mediterranean rivers^41^. As river flows decrease, the resulting river pools are used sequentially by otters, and aggregations similar to the one described in this study occur on a regular annual basis. In addition to this local-scale phenomenon, there are other landscape-scale drivers (e.g. fragmentation) involved in otter population dynamics. It would be interesting to investigate in the future whether this apparently low territoriality is associated with changes in individual fitness and whether higher levels of stress result from the expected increase in individual interactions. The timing—and causes—of aggregation break-up, when otters move into terrestrial environments in search of new pools^27^, should also be studied.

In this study, the number of genotyped individuals was in close agreement with the estimate obtained using the SCR model and was further confirmed by the GoF analysis. The case presented in our study is unusual, as it is rare that more than 60–70% of individuals in the population studied were detected and identified^67^. This could be partly due to our very intensive sampling of this practically closed system of riverine pools. The fit of our model was adequate and we can therefore attribute the heterogeneity in our data to variability in our baseline spraint detections by 'trap'. According to our expectations, sampling effort alone did not sufficiently explain the variability. Probably there were differences between sampling cells (due to amounts of water, vegetation, etc.). Incorporating a random effect on detection rate by trap (cell) was sufficient to achieve an adequate model fit.

In a context of global and rapid human-induced change, the use of methods such as those described above, which explicitly address the detectability of elusive species, allow us to describe ecological response processes that might otherwise go undetected, and to inform management decisions in species conservation with a scientific basis. Our case study of otters suggests a much higher density than previously reported—to the best of our knowledge—under conditions of exceptional prey concentration. Further research is needed to understand when these aggregations break up, what triggers them, and where these animals seek refuge or disperse under conditions of pool drying.

### Supplementary Information

Supplementary Information.

## Data Availability

The datasets generated and/or analyzed during the current study are available in the Zenodo repository at https://zenodo.org/records/10397199.
