# Supplementary material for: Low signs of territorial behavior in the Eurasian otter during low-water conditions in a Mediterranean river
Source: Sci Rep. 2024 May 20;14:11478. doi: 10.1038/s41598-024-62432-1 (PMC11106847; doi:10.1038/s41598-024-62432-1)
Supplement: Supplementary file 1 — Supplementary Information. [file 41598_2024_62432_MOESM1_ESM.docx]

Supplementary Material S1

Low signs of territorial behavior in the Eurasian otter during low-water conditions in a Mediterranean river

José Jiménez^1^. Lucía Del Río^1^. Pablo Ferreras^1^. Raquel Godinho^2.3.4^

^1^ Instituto de Investigación en Recursos Cinegéticos (IREC. CSIC-UCLM-JCCM). Ronda de Toledo 12. 13071 Ciudad Real. Spain.

^2^ CIBIO. Centro de Investigação em Biodiversidade e Recursos Genéticos. InBIO Laboratório Associado. Universidade do Porto. Campus de Vairão. 4485-661 Vairão. Portugal.

^3^ Departamento de Biologia. Faculdade de Ciências. Universidade do Porto. 4169-007 Porto. Portugal.

^4^ BIOPOLIS Program in Genomics. Biodiversity and Land Planning. CIBIO. Campus de Vairão. 4485-661 Vairão. Portugal


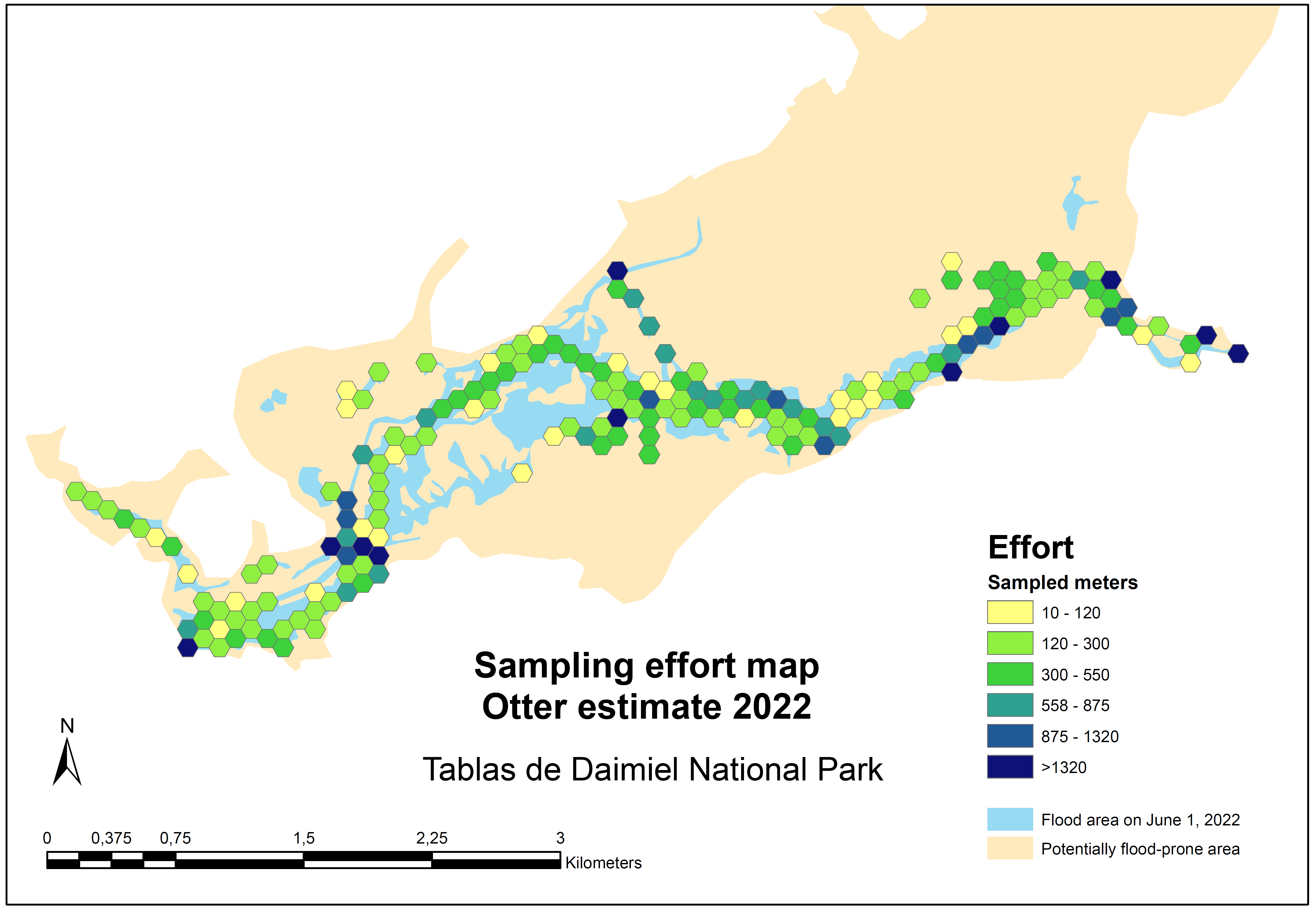


Figure S1. Sampling effort by cell (in meters) in Tablas de Daimiel National Park. Created using ArcGIS.


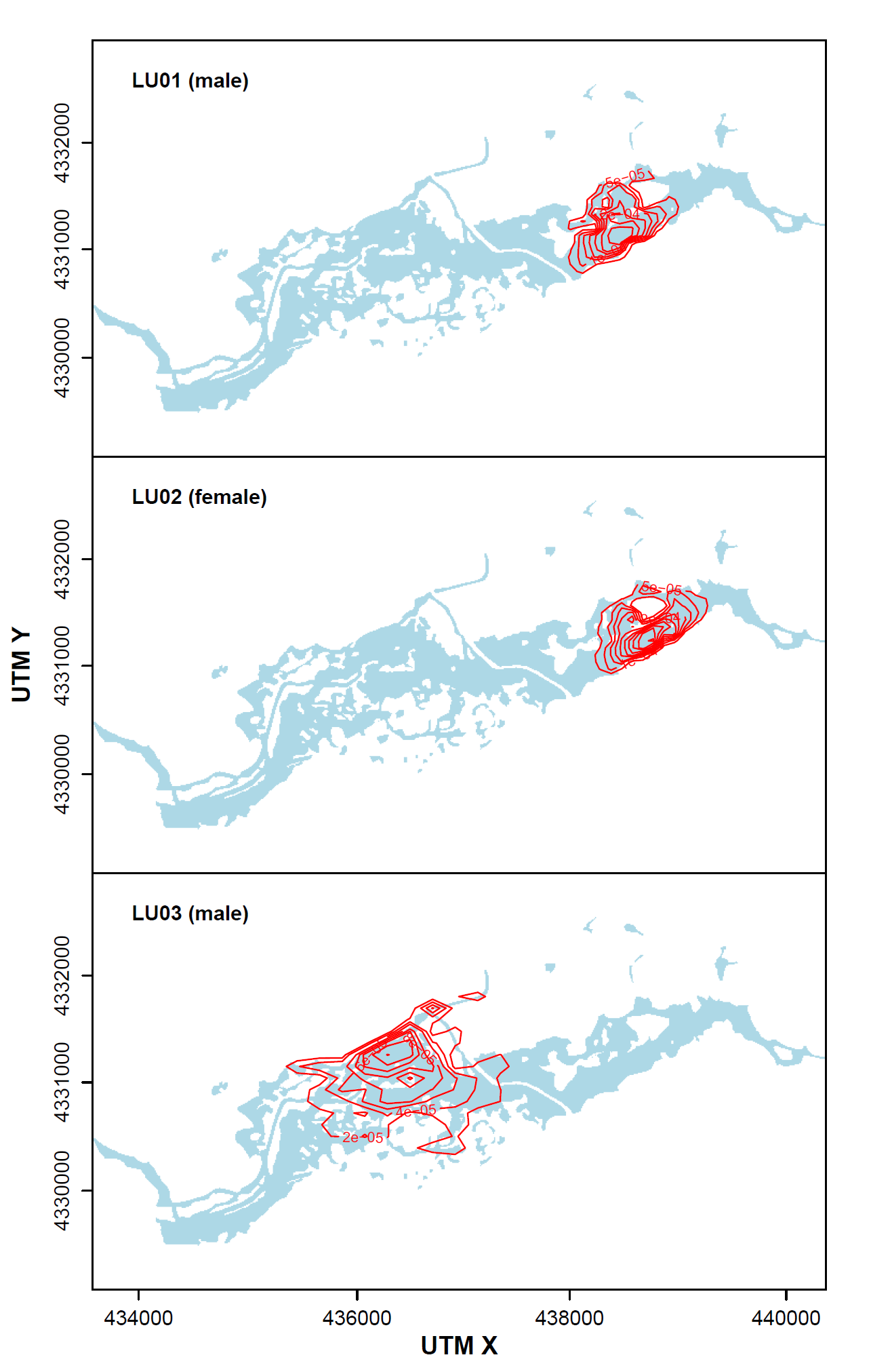


**Figure S2**. Contour maps for the Bayesian posterior probability distribution of the activity centers (Royle et al.. 2014) for individuals 1-3 using the 2D kernel density estimator with the *kde2d* function from MASS (Venables and Ripley. 2002) R (R Core Team. 2023) package.


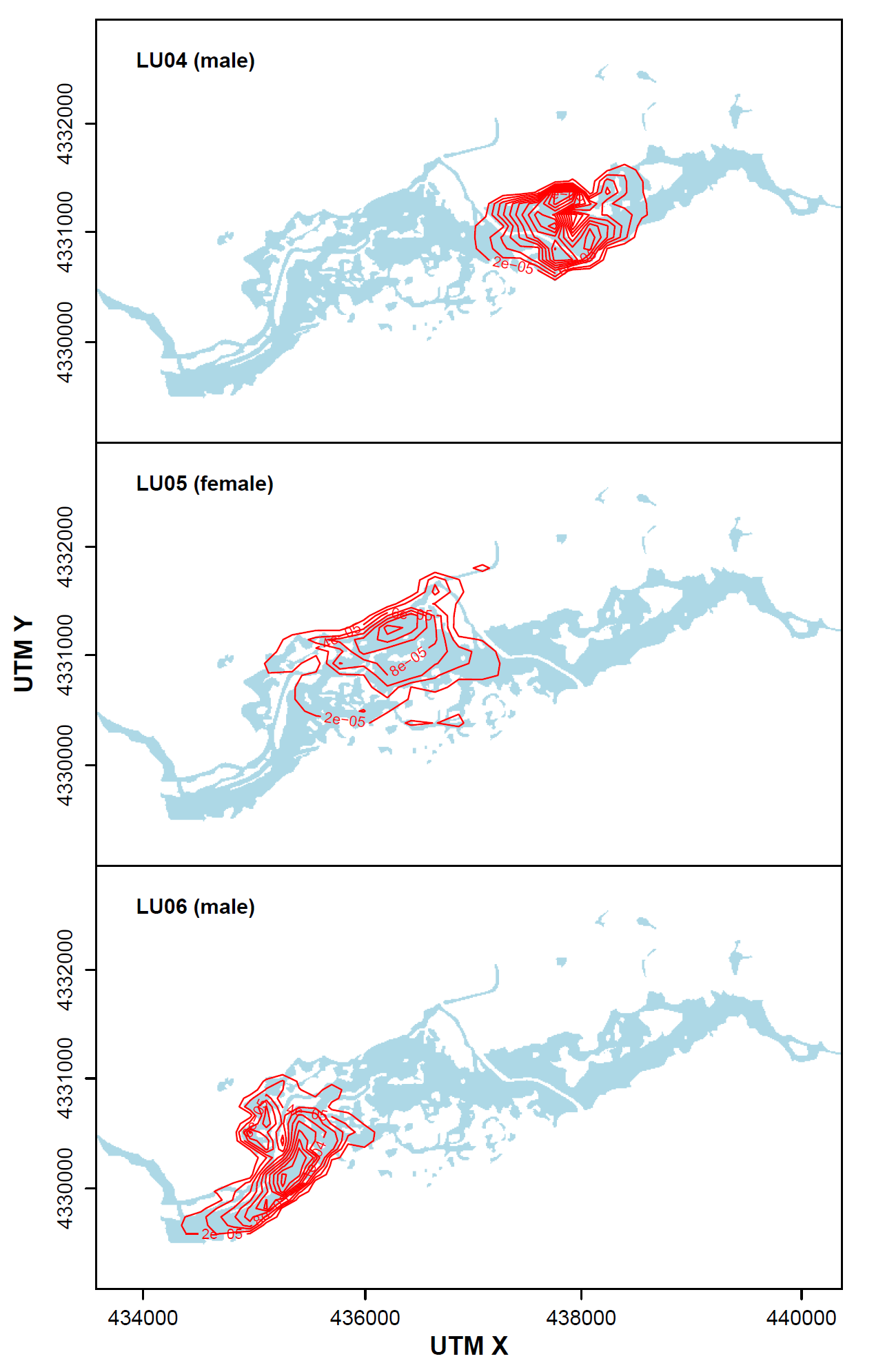


**Figure S3**. Contour maps for the Bayesian posterior probability distribution of the activity centers (Royle et al.. 2014) for individuals 4-6 using the 2D kernel density estimator with the *kde2d* function from MASS (Venables and Ripley. 2002) R (R Core Team. 2023) package.


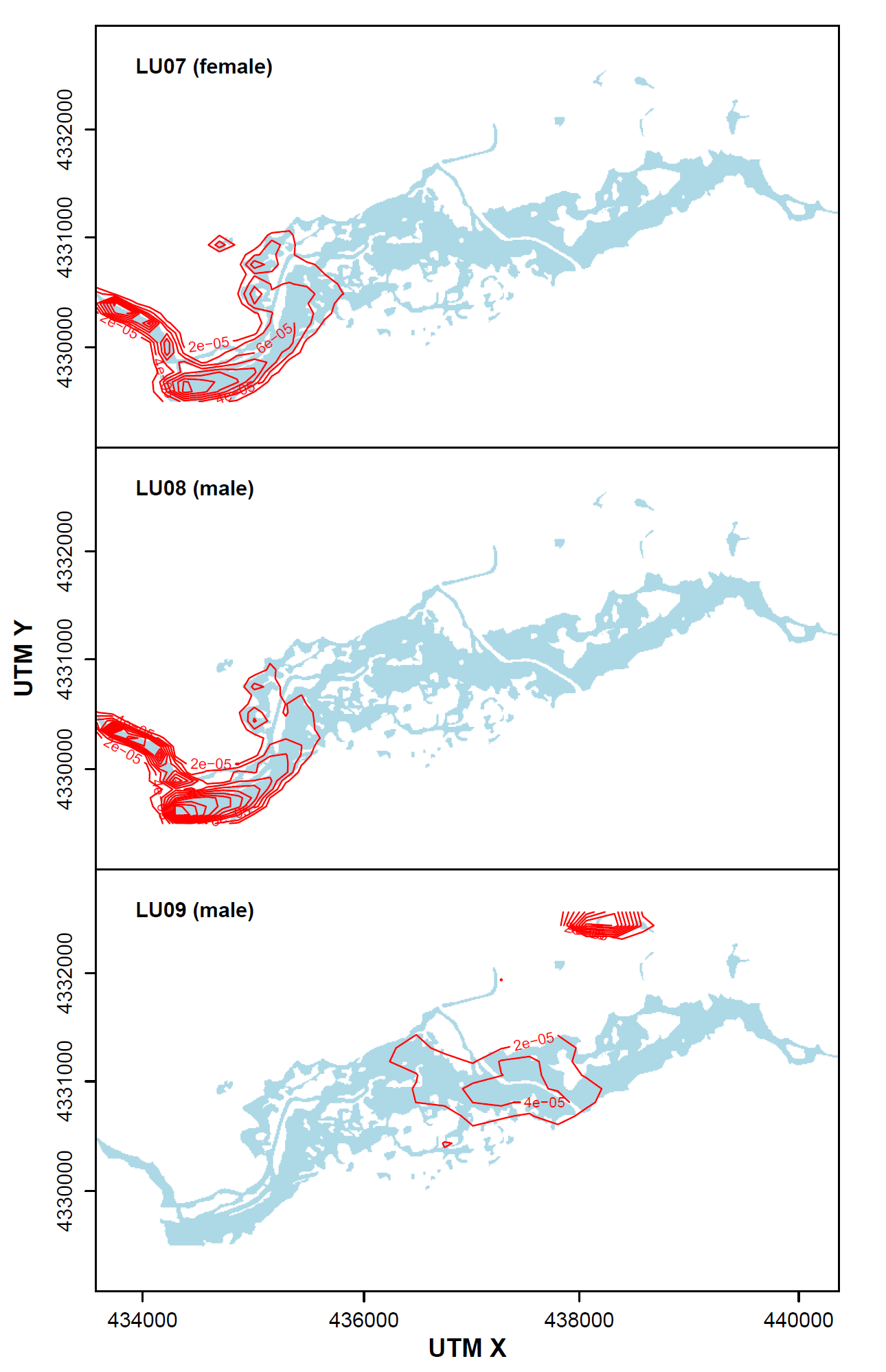


**Figure S4**. Contour maps for the Bayesian posterior probability distribution of the activity centers (Royle et al.. 2014) for individuals 7-9 using the 2D kernel density estimator with the *kde2d* function from MASS (Venables and Ripley. 2002) R (R Core Team. 2023) package.


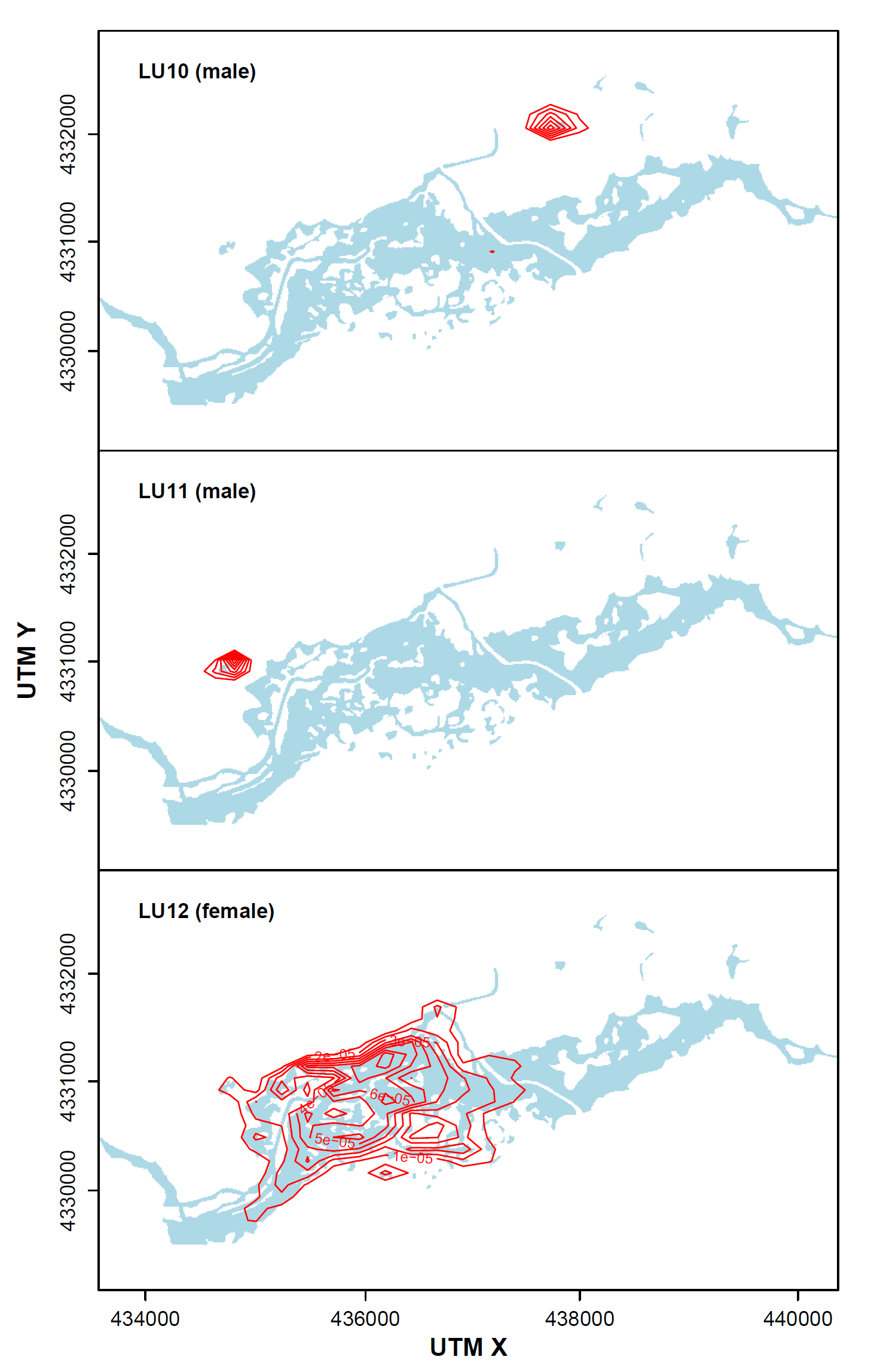


**Figure S5**. Contour maps for the Bayesian posterior probability distribution of the activity centers (Royle et al.. 2014) for individuals 10-12 using the 2D kernel density estimator with the *kde2d* function from MASS (Venables and Ripley. 2002) R (R Core Team. 2023) package.


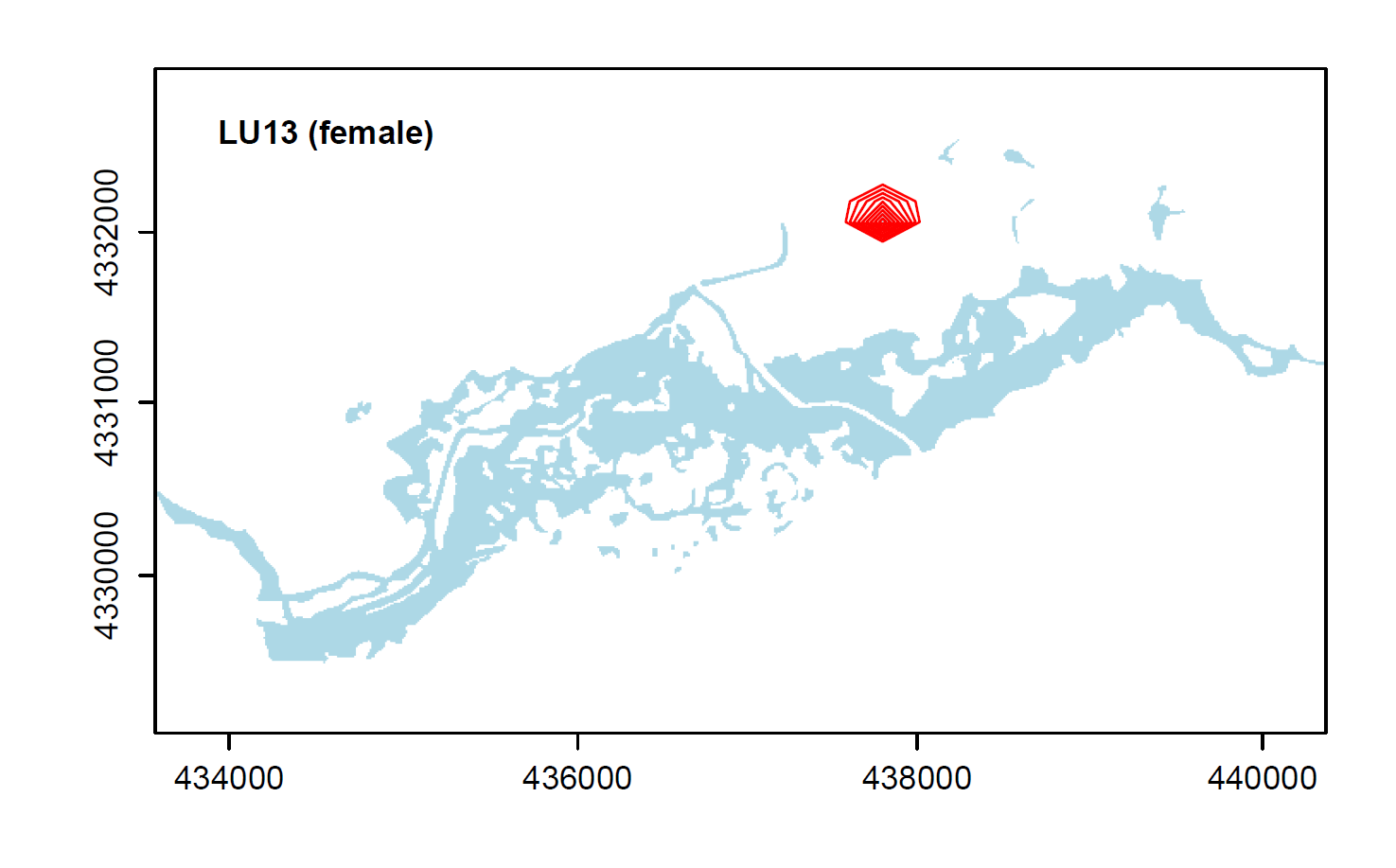


**Figure S6**. Contour maps for the Bayesian posterior probability distribution of the activity centers (Royle et al.. 2014) for individual 13 using the 2D kernel density estimator with the *kde2d* function from MASS (Venables and Ripley. 2002) R (R Core Team. 2023) package.

**Table S1**. Nuclear markers used for individual and sex identification of otters. The multiplex in which markers were amplified, respective concentration and dye in the multiplex, and the original reference of each marker are indicated.

| **Markers** | **Usage** | **Dye** | **Concentration (pM)** | **Multiplex mix** | **Reference** |
| --- | --- | --- | --- | --- | --- |
| Lut435 | Individual ID | VIC | 0.8 | MIX1 | Dallas & Piertney 1998 |
| Lut453 | Individual ID | VIC | 0.8 | MIX2 | Dallas & Piertney 1998 |
| Lut457 | Individual ID | PET | 1.2 | MIX1 | Dallas & Piertney 1998 |
| Lut604 | Individual ID | FAM | 0.6 | MIX2 | Dallas & Piertney 1998 |
| Lut615 | Individual ID | PET | 2.0 | MIX2 | Dallas & Piertney 1998 |
| Lut701 | Individual ID | FAM | 0.6 | MIX3 | Dallas & Piertney 1998 |
| Lut715 | Individual ID | VIC | 0.8 | MIX3 | Dallas & Piertney 1998 |
| Lut717 | Individual ID | NED | 0.6 | MIX1 | Dallas & Piertney 1998 |
| Lut733 | Individual ID | VIC | 0.8 | MIX4 | Dallas & Piertney 1998 |
| Lut782 | Individual ID | NED | 0.8 | MIX4 | Dallas & Piertney 1998 |
| Lut818 | Individual ID | VIC | 1.2 | MIX3 | Dallas & Piertney 1998 |
| Lut832 | Individual ID | NED | 0.8 | MIX3 | Dallas & Piertney 1998 |
| Lut833 | Individual ID | NED | 0.6 | MIX2 | Dallas & Piertney 1998 |
| OT02 | Individual ID | NED | 0.8 | MIX1 | Huang et al. 2005 |
| OT04 | Individual ID | PET | 1.2 | MIX3 | Huang et al. 2005 |
| OT05 | Individual ID | PET | 1.2 | MIX4 | Huang et al. 2005 |
| OT07 | Individual ID | FAM | 1.0 | MIX4 | Huang et al. 2005 |
| OT14 | Individual ID | FAM | 0.6 | MIX1 | Huang et al. 2005 |
| OT17 | Individual ID | FAM | 0.8 | MIX3 | Huang et al. 2005 |
| OT22 | Individual ID | FAM | 0.8 | MIX2 | Huang et al. 2005 |
| LutSRY | Sex ID | FAM | 0.8 | MIX1 | Dallas et al. 2000 |

**Table S2**. Thermocycling conditions for the amplification of the four mutiplex reactions (same conditions for all) encompassing the 21 markers used in the analysis of otters’ non-invasive samples. Final volume for all reactions = 10ul. AT = Annealing temperature; ET = Extension temperature.

| **Denaturation temperature/time** | **AT/time**  **1^st^ PCR** | **AT/time**  **2^nd^ PCR** | **ET/time** | **Final ET/time** |
| --- | --- | --- | --- | --- |
| 95°C/15 min | 62°-58° (-0.5°C/cycle)  60 sec  9 cycles  +  58°C  60 sec  11 cycles | 62°-58° (-0.5°C/cycle)  60 sec  9 cycles  +  58°C  60 sec  31 cycles | 72°C/30 sec | 60°C/30 min |

**Table S3**. Amplification rates and genotyping errors (non-amplification of an allele -dropout- and appearance of false alleles) by molecular marker.

| **Loci** | **Amplification rate** | **Dropout rate** | **False alleles rate** |
| --- | --- | --- | --- |
| Lut435 | 0.94 | 0.184 | 0 |
| Lut453 | 0.94 | 0.397 | 0 |
| Lut457 | 0.95 | 0.399 | 0 |
| Lut604 | 0.97 | 0.268 | 0 |
| Lut615 | 0.91 | 0.207 | 0 |
| Lut701 | 0.87 | 0.420 | 0.003 |
| Lut715 | 0.89 | 0.491 | 0 |
| Lut717 | 0.86 | 0.590 | 0 |
| Lut733 | 0.93 | 0.532 | 0.023 |
| Lut782 | 0.87 | 0.409 | 0.025 |
| Lut818 | 0.85 | 0.495 | 0 |
| Lut832 | 0.87 | 0.533 | 0 |
| Lut833 | 0.91 | 0 | 0 |
| OT02 | 0.81 | 0 | 0 |
| OT04 | 0.87 | 0.613 | 0 |
| OT05 | 0.91 | 0.429 | 0 |
| OT07 | 0.93 | 0.356 | 0 |
| OT14 | 0.94 | 0.250 | 0.002 |
| OT17 | 0.97 | 0.344 | 0 |
| OT22 | 0.97 | 0.401 | 0.003 |
| **Average** | **0.91** | **0.366** | **0.003** |

**Table S4.** Probability of Identity (PID) and Probability of Identity among siblings (PIDsib) for otters in the Tablas de Daimiel National Park calculated for 10 datasets of 14 randomly selected loci across our 20 loci dataset.

| **dataset** | **PID** | **PIDsib** |
| --- | --- | --- |
| 1 | 7.69E-07 | 1.38E-03 |
| 2 | 7.18E-06 | 3.44E-03 |
| 3 | 5.26E-07 | 1.09E-03 |
| 4 | 7.37E-07 | 1.57E-03 |
| 5 | 2.36E-07 | 8.37E-04 |
| 6 | 2.09E-07 | 6.55E-04 |
| 7 | 6.11E-07 | 1.46E-03 |
| 8 | 1.11E-07 | 6.65E-04 |
| 9 | 3.26E-07 | 9.95E-04 |
| 10 | 9.93E-07 | 1.28E-03 |


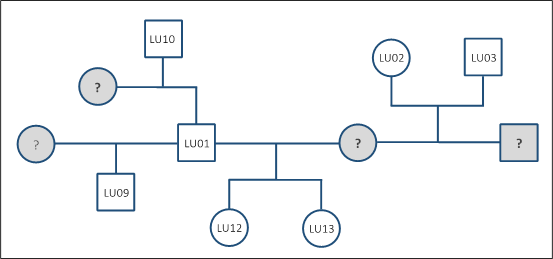


**Figure S7.** Genealogy of the seven otters inferred to have a direct familial relationship. Horizontal lines connect parents, and vertical lines indicate offspring. Unsampled parents are depicted in grey. Squares and circles indicate males and females, respectively.

REFERENCES

Dallas JF, Carss DN, Marshall F, Koepfli KP, Kruuk H, Bacon PJ, Piertney SB (2000). Sex identification of the Eurasian otter Lutra lutra by PCR typing of spraints. Conservation Genetics, 1(2), 181-183.

Dallas JF, Piertney SB (1998). Microsatellite primers for Eurasian otter. Molecular Ecology, 7, 1247-1251.

Esri. ArcGIS Desktop: Release 10.8. (2020).

Huang CC, Hsu YC, Lee LL, Li SH (2005). Isolation and characterization of tetramicrosatellite DNA markers in the Eurasian otter (Lutra lutra). Molecular Ecology, 5, 314-316.

R Core Team. 2023. R: A language and environment for statistical computing. R Found. Stat. Comput.

Royle. J.A.. Chandler. R.B.. Sollmann. R.. Gardner. B.. 2014. Spatial capture-recapture. Elsevier. Academic Press. Waltham. Massachusetts. https://doi.org/10.1016/B978-0-12-405939-9.00026-8

Venables. W.N.. Ripley. B.D.. 2002. Modern Applied Statistics with S. Fourth. ed. Springer. New York. NY.
